# Automatic Segmentation of Metastatic Livers by Means of U-Net-Based Procedures

**DOI:** 10.3390/cancers16244159

**Published:** 2024-12-13

**Authors:** Camilla Tiraboschi, Federica Parenti, Fabio Sangalli, Andrea Resovi, Dorina Belotti, Ettore Lanzarone

**Affiliations:** 1 Department of Management, Information and Production Engineering, University of Bergamo, 24044 Dalmine, BG, Italy; 2 Department of Biomedical Engineering, Istituto di Ricerche Farmacologiche Mario Negri IRCCS, 24126 Bergamo, BG, Italy; 3 Department of Oncology, Istituto di Ricerche Farmacologiche Mario Negri IRCCS, 24126 Bergamo, BG, Italy

**Keywords:** pancreatic ductal adenocarcinoma, micro-CT, liver metastases, automatic segmentation, U-net

## Abstract

**Background:** The liver is one of the most common sites for the spread of pancreatic ductal adenocarcinoma (PDAC) cells, with metastases present in about 80% of patients. Clinical and preclinical studies of PDAC require quantification of the liver’s metastatic burden from several acquired images, which can benefit from automatic image segmentation tools. **Methods:** We developed three neural networks based on U-net architecture to automatically segment the healthy liver area (HL), the metastatic liver area (MLA), and liver metastases (LM) in micro-CT images of a mouse model of PDAC with liver metastasis. Three alternative U-nets were trained for each structure to be segmented following appropriate image preprocessing and the one with the highest performance was then chosen and applied for each case. **Results:** Good performance was achieved, with accuracy of 92.6%, 88.6%, and 91.5%, specificity of 95.5%, 93.8%, and 99.9%, Dice of 71.6%, 74.4%, and 29.9%, and negative predicted value (NPV) of 97.9%, 91.5%, and 91.5% on the pilot validation set for the chosen HL, MLA, and LM networks, respectively. **Conclusions:** The networks provided good performance and advantages in terms of saving time and ensuring reproducibility.

## Simple Summary

In this work, we developed three neural networks based on the U-net architecture to automatically segment the healthy liver area, the metastatic liver area, and liver metastases in micro-CT images of mice with pancreatic ductal adenocarcinoma and liver metastases. The best network for each task was then identified by cross-validation. The results demonstrated the ability of the selected networks to segment the above areas in a manner comparable to manual segmentation, at the same time saving time and ensuring reproducibility. Therefore, despite the limited number of animals involved, our pilot study represents a first step toward the development of automated tools to support liver metastasis research in the preclinical setting.

## 1. Introduction

The liver is a common site for metastases of many primary cancer types, such as pancreatic, colorectal, lung and breast cancers, and melanoma [1]. In particular, metastases contribute strongly to the mortality of patients with pancreatic ductal adenocarcinoma (PDAC), with up to 80% of patients with PDAC developing liver metastases [2]. Tumor cell dissemination is an early event during PDAC progression; however, since most patients have no symptoms and have already been diagnosed with advanced metastatic disease [3], only 20% are eligible for surgical treatment. Indeed, the likelihood of patient recovery is related to the stage at which PDAC is diagnosed. The earlier the diagnosis, the greater the chances that patients are operated on and respond to treatment. In this scenario, tools for the automatic quantification of liver metastatic burden in different CT images play a key role. They make it possible to study the molecular mechanisms underlying the development of liver metastases, evaluate antimetastatic therapies, and identify biomarkers of tumor progression.

To date, manual slice-by-slice image segmentation has mainly been used, but it has two main disadvantages: it is time-consuming and extremely operator-dependent, since it is performed by a trained biologist or physician. Available semiautomated segmentation techniques can help solve some of the problems associated with traditional manual segmentation. They reduce inter and intraobserver variability and save highly trained professionals’ time and effort. Even better than this, artificial intelligence techniques could be exploited for automatic segmentation of liver images. In this field, convolutional neural networks (CNNs) have recently demonstrated the ability to provide fully automated segmentations with outstanding performance in various image segmentation tasks, such as the semantic segmentation of medical images. In particular, the U-net, which consists of a contracting path and an expanding path, has been widely used since it was proposed in 2015 [4] in a variety of heterogeneous problems [5,6].

Seo et al. [7] proposed a modified U-net that adaptively incorporates features in the residual path into features in the skip connection and applied it to the public dataset of the 2017 Liver Tumor Segmentation Challenge (LiTS17). Ayalew et al. [8] proposed a modified U-net with a batch normalization after each convolutional layer and a dropout layer after each convolutional block of the contracting path and applied it to segment livers and tumors in abdominal CT images. Gong et al. [9] proposed an architecture with one U-net used to obtain the approximate position of the liver and tumor and a second U-net used for accurate segmentation of the actual liver tumors in the region obtained by the first. They added residual modules and dense connections to the U-nets and introduced multidimensional information fusion. Manjunath and Kwadiki [10] developed a U-net with 58 layers to segment livers and tumors in CT images and tested their approach on the 3D-IRCADb dataset. Danciu et al. [11] used a modified U-net for automatic segmentation of liver and hepatic tumors, which includes three downsampling blocks, a central concatenated dilated bottleneck, and three upscaling blocks and applied it to the 3D-IRCADb-01 dataset. Aparna and Libish [12] addressed the automatic segmentation and classification of hepatic tumors in CT images using a dense U-net architecture that includes dense blocks as core elements, consisting of straight connections from every layer to all subsequent layers. Saumiya and Franklin [13] introduced a multitask network for combined automatic liver tumor segmentation and classification. Focusing on segmentation, they proposed a U-net with an attention-based deformable module instead of convolution and a residual skip connection.

The aim of this work is to propose and validate a set of CNNs based on U-net architecture to segment the liver and its metastases in images related to preclinical studies conducted in animals. In particular, we refer to micro-CT images of livers obtained from mice with PDAC. Due to the specific geometric characteristics of metastases, which may differ from those of primary tumors, we believe that it is necessary to train ad hoc networks for liver metastases, which is the purpose of our work. This idea has been confirmed by other studies, which were specifically devoted to metastases in several fields [14].

**Figure 1:**
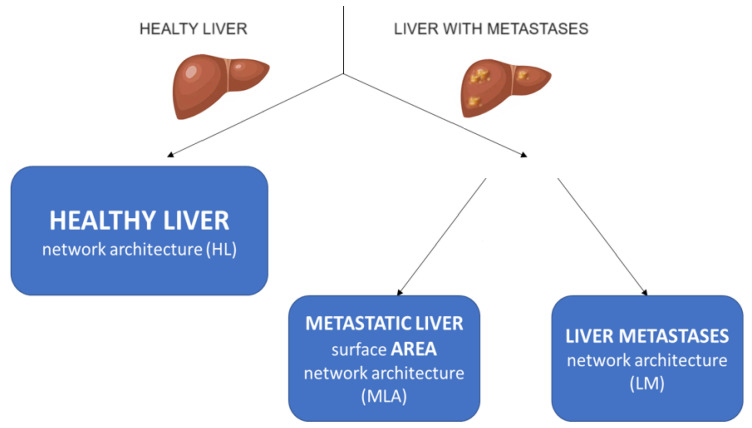
Proposed architecture consisting of three CNNs: the healthy liver (HL) network, the metastatic liver area (MLA) network, and the liver metastases (LM) network.

To preserve the specificity of the different segmentations required, we developed three networks that segment the healthy liver area, the metastatic liver area, and the liver metastases. Hereafter, these networks are referred to as the healthy liver (HL) network, the metastatic liver area (MLA) network, and the liver metastases (LM) network (Figure 1). This differentiation allows several specialized CNNs to be considered in light of the complexity of the segmentation problems and the inefficiency of considering a single global CNN that can address all cases. At the same time, it allows for the creation of a practical tool that can be used in a variety of cases. This is in agreement with the literature, in which architectures built by different networks have been reported for complex tasks [15,16].

Three alternative CNN structures were trained for each of the HL, MLA, and LM cases. In each case, the structure with the highest performance was chosen and applied.

CNNs have hardly been applied to the segmentation of metastases in the liver. To the best of our knowledge, only Fehrenbach et al. [17] applied the open-source MIC-DKFZ nn-Net to segment liver metastases, along with liver parenchyma and neuroendocrine neoplasms, but from MRI images. Applications in the case of metastasis usually include other types of studies; for example, Trivizakis et al. [18] used a network consisting of four consecutive 3D convolutional layers and rectified linear unit (ReLU) activation function, followed by a fully connected layer and a Softmax layer for binary classification. The goal was to discriminate between primary and metastatic tumors in the liver using diffusion-weighted magnetic resonance imaging data.

Therefore, this work can be considered a pilot study, and its contributions lie in the different CNNs, in the architecture itself, and in addressing the segmentation problem of PDAC metastases in the liver, which is still an open issue even at the level of preclinical studies. We are aware of future extensions that will make the proposed architecture and networks robust, especially in terms of including more mice with PDAC, but we believe that this work represents an advance over the literature on automatic segmentation of metastases in the liver.

## 2. Materials and Methods

### 2.1. Murine Model

FC1199 cells, kindly provided by D.A. Tuveson (Cold Spring Harbor, NY, USA) [19], were derived from tumors arisen in LSL-Kras^G12D/+^; LSL-Trp53^R172H/+^; Pdx-1-Cre mice in the C57BL/6 background. Cells were grown in Dulbecco’s modified Eagle medium (Gibco, ThermoFisher Scientific, Rodano, MI, Italy) supplemented with 10% FBS (Euroclone, Milan, Italy) and 1% L-glutamine (Gibco, ThermoFisher Scientific, Rodano, MI, Italy). Artificial metastases were obtained by injecting $5\cdot 10^{4}$. FC1199 cells in the spleen of 10-week-old female C57BL/6 mice (Envigo, Correzzana, MB, Italy) [20]. The mice were kept under pathogen-free conditions, housed in isolated and ventilated cages, and treated with aseptic procedures. They were then subjected to CT analysis to assess the presence of liver metastases and then euthanized. After sacrifice, the presence of liver metastases was confirmed by counting macroscopic superficial nodules. An FC1199-bearing mouse and a healthy mouse were included in this pilot study.

### 2.2. Image Acquisition and Preprocessing

For CT scanning, 100 μL of ExiTron^TM^ nano 6000 (Miltenyi Biotec GmbH, Bergisch Gladbach, Germany) was perfused into the tail vein of the mice 10 days after tumor transplantation [21]. This contrast agent, used specifically for preclinical CT, is an alkaline-earth metal-based nanoparticulate, which is visible from 2 h after injection up to 30 days. It is internalized by macrophages in the liver and spleen; consequently, these organs are illuminated by the contrast nanoparticles due to the 1-month turnover of macrophages [21]. Liver specimens were then scanned with micro-CT at different times after contrast medium injection. Our images refer to acquisition on day 21 after ExiTron^TM^ nano 6000 injection.

**Figure 2:**
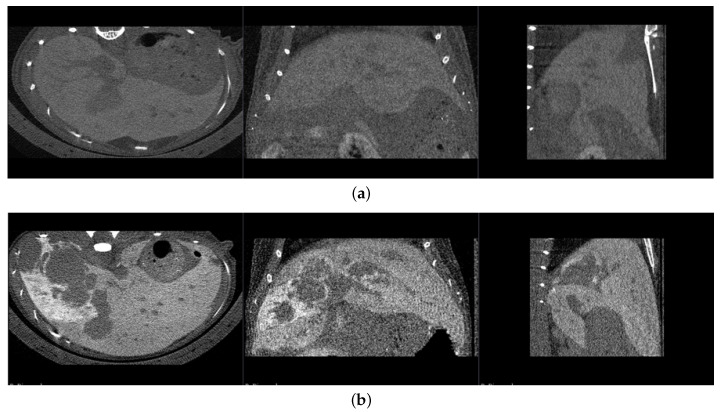
Healthy liver slice (**a**) and metastatic liver slice (**b**) in the sagittal (∼2600 × 1500 pixels), frontal (∼2600 × 1600 pixels), and transverse planes (∼1500 × 1600 pixels), visualized with open source software 3DSlicer (version 5.7) [22].

The images were acquired using the SkyScan 1076 (Bruker, Billerica, MA, USA), a high-performance in vivo machine with an X-ray detector of 4000×2300 pixels and a spatial resolution of up to 9 μm pixel size. They were acquired at maximum resolution (9μm in each principal direction) with the following parameters: source voltage of 50 kV, source current of 100 μA, filter of 0.5 mm Al, exposure of 1200 ms, rotation step of 0.8 deg (250 images), and scan duration of 15 min. Volume images were reconstructed with a backprojection algorithm implemented using NRecon software (version 1.7.4.6, Bruker, Billerica, MA, USA). Examples of slices from healthy and metastatic livers are shown in Figure 2.

The image resolution was first reduced by a factor of 1:2 in the third dimension (resulting in 18 μm) by retaining one slice out of two. This was achieved using DataViewer software (Bruker, Billerica, MA, USA) to reduce the computational effort required to train the networks. Next, a median filter of 2 pixels was applied using software ImageJ (version 1.54f) [23]. These operations were performed to make the training of CNNs feasible in terms of computational effort and occupied memory while preserving image features in terms of liver and metastases.

Stacks were exported in the nearly raw raster data (NRRD) format. Moreover, the following preprocessing steps were applied to the images: (*i*) resampling: images were read as gray-scale figures reshaped to a single channel; (*ii*) resizing: images were resized into squared images with a size of 512×512 pixels by adding a black border around them; and (*iii*) blurring: 5×5 Gaussian blurring was applied to the images. Resizing images into a square shape is necessary when dealing with CNNs using TensorFlow (see Section 2.4) for several reasons, which include computational efficiency, consistency of input dimensions, and the architectural requirements of CNNs. Gaussian blurring is a preprocessing technique commonly used to clean up radiographic images, making them more suitable for later processing, such as segmentation, because it reduces noise without decreasing detail and makes the edges of structures better defined. The specific choice of a 5×5 grid was evaluated by visual analysis by an experienced operator.

Finally, segmentation by the MLA and LM networks was preceded by low-effort manual cleaning of tissues easy to identify and completely disjointed from the area to be recognized. Specifically, spleen and bone were removed manually using 3DSlicer [22] and ImageJ [23] software. Consistently, these tissues were also removed during the creation of the ground truths (GTs).

### 2.3. Dataset and Ground Truths

The database of our pilot study consisted of 740 healthy liver slices and 700 liver slices with metastases, which were from two mice.

The available images of the healthy liver and the liver with metastases were randomly divided into a training and a validation set. For the healthy liver images (HL network) 80% of the images were entered into the training set and 20% into the validation set, corresponding to 592 and 148 images in the training and validation sets, respectively. For the metastatic liver images (MLA and LM networks), 70% of the images were entered into the training set and 30% into the validation set, corresponding to 490 and 210 images in the training and validation set, respectively. These numbers are in line with those reported in other studies dealing with training CNNs [10,24].

Segmentations for GTs were obtained by a semiautomatic approach performed using software 3DSlicer (version 5.7) [22], which ensured the adequacy and repeatability of the process. First, a presegmentation of the livers and metastases was manually performed using the Segment Editor software module, which consists of segmenting the area of the liver (healthy and metastatic) and liver metastases in each fifth slice in the axial direction of the total volume. Subsequently, the masks of the remaining slides were obtained automatically by adding segmentations statistically similar to those preinserted using the Fill-in software option.

### 2.4. Networks

Image screening was first performed to determine whether the acquired images belonged to a healthy or metastatic liver; currently, this is still a manual operation, but it is quick and easy to perform with little inter and intraoperator variability. Then, each CT scan was routed to the HL network or the MLA and LM networks, depending on the screening result, where the actual segmentation activity took place.

The structures of the CNN variants for HL, MLA, and LM are reported in Table 1. They are referred to as U-net-1, U-net-2 and U-net-3, respectively. In agreement with [6], the blocks reported in the table are as follows:

- Block 1 is the concatenation of (*i*) the sequence of a 1×1 convolution and a batch normalization; (*ii*) the sequence of a 3×3 convolution and a batch normalization; (*iii*) the sequence of a 1×1 convolution and a batch normalization; (*iv*) the sequence of a 1×1 convolution and a batch normalization; and (*v*) the sequence of a 2×2 max pooling and a 1×1 convolution.
- Block 2 is the concatenation of (*i*) the sequence of a 1×1 convolution and a batch normalization; (*ii*) the sequence of a 3×3 convolution, a batch normalization, a 3×3 convolution, and a batch normalization; (*iii*) the sequence of a 1×1 convolution, a batch normalization, a 3×3 convolution, and a batch normalization; (*iv*) the sequence of a 1×1 convolution and a batch normalization; and (*v*) the sequence of a 2×2 max pooling and a 1×1 convolution.
- Block 3 is the concatenation of (*i*) the sequence of a 1×1 convolution and a batch normalization; (*ii*) the sequence of a 3×3 convolution, a batch normalization, a 3×3 convolution, and a batch normalization; (*iii*) the sequence of a 1×1 convolution, a batch normalization, a 3×3 convolution, a batch normalization, a 3×3 convolution, and a batch normalization; (*iv*) the sequence of a 1×1 convolution and a batch normalization; and (*v*) the sequence of a 2×2 max pooling and a 1×1 convolution.

In all alternatives, a Sigmoid activation function was applied to the final layer and a Leaky ReLu activation function was applied to every other neuron. In this way, the outcome for each pixel was in terms of the probability to be associated with the considered label. The obtained probabilities were finally binarized through a thresholding operation using a fixed threshold τ to provide the binarized predicted mask (BPM). Each pixel was classified as positive if a probability greater than τ was obtained in it and vice versa. This also allowed to obtain the confusion matrix for cross-validation and calculate different performance metrics.

These alternative networks were chosen because they represent common U-net architectures used in the literature, especially for medical imaging [4,25,26,27,28]. The goal was to take alternatives from those validated in the literature for similar problems and determine the most effective one for each of the three tasks addressed in our work (HL, MLA, and LM).

The hyperparameters for the different specialized networks are detailed in Table 2. Given a specialized network (HL, MLA, or LM), each network structure (U-net-1, U-net-2, and U-net-3) had the same hyperparameters. These values were empirically selected for each network structure independently to ensure an optimal tradeoff between high accuracy, rapid convergence, lower overfitting, and higher generalization. All networks were trained using the binary cross-entropy loss function.

The networks were coded in Python (version 3.7.6), using the TensorFlow (version 2.6.4) deep learning library integrated with Keras API, and implemented using Kaggle. Data augmentation was applied to the images in the training set and the corresponding GTs. The following modifications were applied: width and shift range of 0.1, horizontal and vertical flipping, and zooming in the range of [0.9,1.1]. Augmentation was performed while training the models at each epoch by means of the flow_from_directory method available in Keras.

### 2.5. Evaluation Metrics

Performance assessment was based on the confusion matrix between BPM and GT, which includes the number of true positive (TP), true negative (TN), false positive (FP), and false negative (FN) pixels. The following metrics were then derived from these four indices:

- *Accuracy*: the rate of pixels correctly classified:
  $$Accuracy=\frac{TP+TN}{TP+FP+TN+FN}.$$
- *Specificity*: the rate of true negatives over all negative pixels in the GT:
  $$Specificity=\frac{TN}{TN+FP}.$$
- *Precision*: the true positive rate:
  $$Precision=\frac{TP}{TP+FP}.$$
- *Negative Predicted Value* (NPV): the true negative rate:
  $$NPV=\frac{TN}{TN+FN}.$$
- *Recall*: the rate of true positives over all positive pixels in the GT:
  $$Recall=\frac{TP}{TP+FN}.$$
- Intersection over union (IoU): the relative overlap between BPM and GT, computed as the area of overlap divided by the area of union between BPM and GT:
  $$IoU=\frac{\mathrm{BPM}\;\cap \;\mathrm{GT}}{\mathrm{BPM}\;\cup \;\mathrm{GT}}=\frac{TP}{TP+FN+FP}.$$
  This has a range of [0,1], where zero means no overlap and one perfect overlap.
- *Dice*: twice the overlap area divided by the total number of positive pixels in both images:
  $$Dice=2\;\frac{\left|\mathrm{BPM}\;\cap \;\mathrm{GT}\right|}{\left|\mathrm{GT}|+|\mathrm{BPM}\right|}=\frac{2\;TP}{2\;TP+FN+FP}.$$
  This is equivalent to the *F1* score, defined as twice the harmonic mean of *Precision* and *Recall*.

## 3. Results

The binarization threshold τ was set to 5/255 for all HL, MLA, and LM networks. This value was chosen because it provides the best performance. It is low because, in all cases, low probabilities were associated with the pixels of the structure of interest. With this threshold, we obtained the metrics reported in Table 3 on the validation set.

### 3.1. HL Networks

The results in Table 3 show good accuracy and specificity of the HL networks, with adequate precision and recall.

Comparing U-net-1, U-net-2, and U-net-3, none of them outperformed in all metrics. Therefore, a tradeoff analysis was performed based on a combination of the highest NPV, IoU, and Dice coefficients and the visual inspection of some predicted masks. In this view, U-net-2 showed the highest NPV, while U-net-1 and U-net-3 performed better in terms of IoU and Dice coefficients.

**Figure 3:**
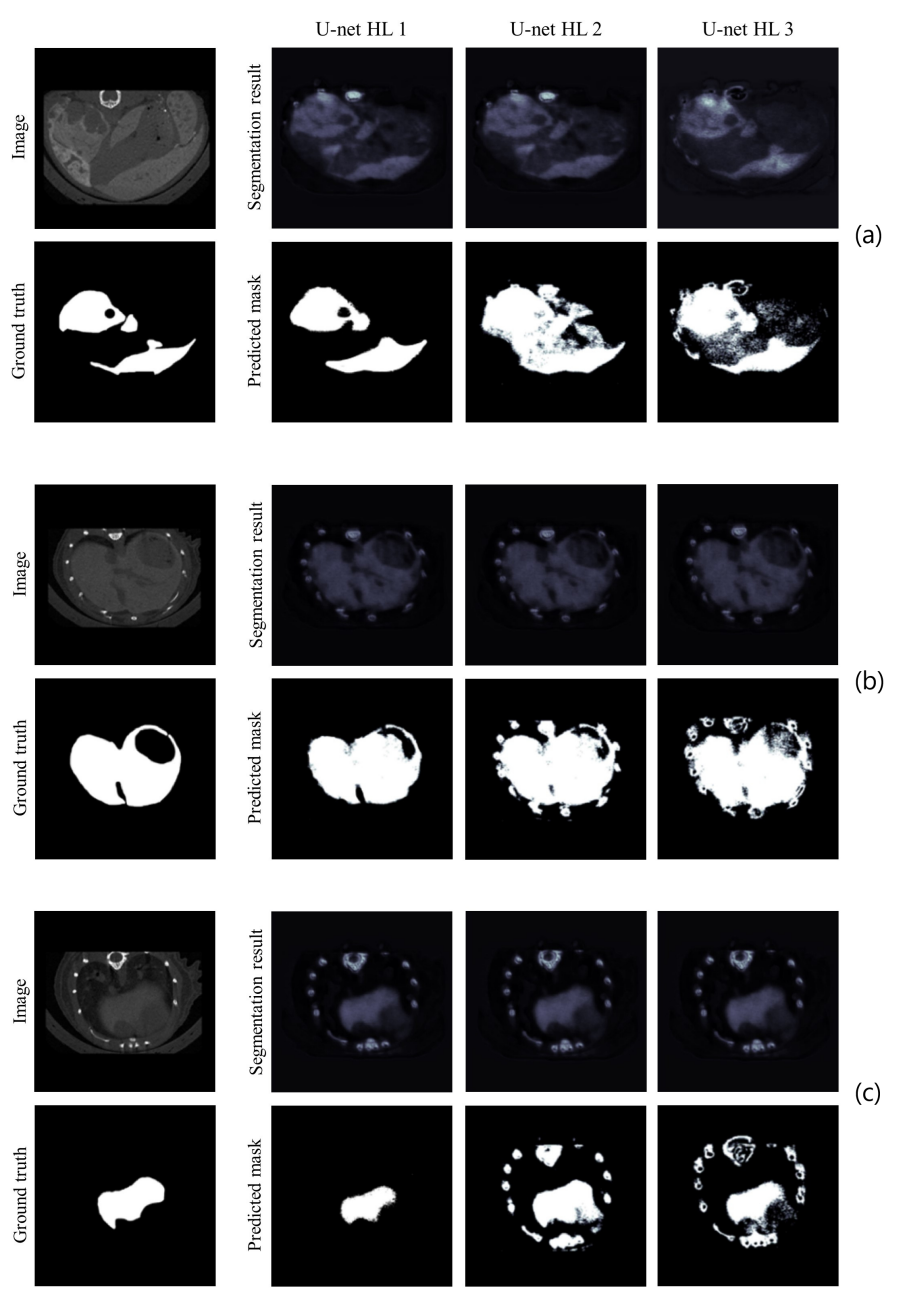
Sample images of healthy liver for HL: proximal slice (**a**), slice in the middle (**b**), and distal slice (**c**). The acquired image, the corresponding GT, the HL segmentations before binarization, and the predicted BPMs from all networks (U-net-1, U-net-2, and U-net-3) are reported for each slice.

For the visual inspection, Figure 3 shows some sample images from the validation set, the corresponding GT, the segmentation results before binarization, and the BPM. Figure 3a refers to a proximal slice of the liver, Figure 3b to a central slice, and Figure 3c to a distal slice. It can be seen that U-net-1 segments the considered slices better, recognizing the correct shape of the liver. In addition, it is the only model that correctly excludes vertebrae and ribs. This feature offers U-net-1 the ability to perform well in HL segmentation, making it the best model among the three alternatives.

### 3.2. MLA Networks

The evaluation of the metastatic liver is more challenging due to the alterations induced by the metastases. In any case, Table 3 shows that the MLA networks have good accuracy and specificity comparable with those of HL, namely slightly lower accuracy but slightly better specificity. The other metrics are also in line with those of HL, with lower NPV and recall, but higher IoU and Dice, especially for U-net-2 and U-net-3.

**Figure 4:**
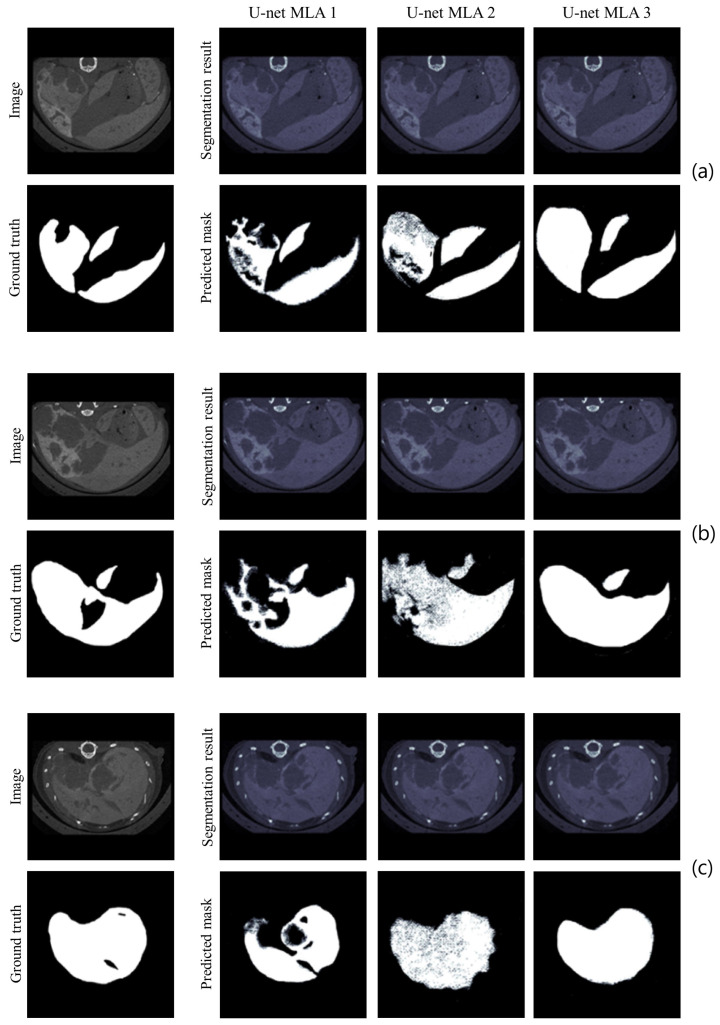
Sample images of liver with metastases for MLA: proximal slice (**a**), slice in the middle (**b**), and distal slice (**c**). The acquired image, the corresponding GT, the MLA segmentations before binarization and the predicted BPMs from all networks (U-net-1, U-net-2, and U-net-3) are reported for each slice.

Again, none of the models outperformed in all metrics, and the same tradeoff analysis was performed as in the HL case. U-net-2 and U-net-3 performed best in terms of IoU and Dice coefficients. As for the visual inspection, Figure 4 shows some sample images of the validation set, the corresponding GT, the segmentation result before binarization, and the BPM. U-net-3 is shown to provide the maps closest to the GT, correctly segmenting the whole liver area on the different slices, which makes it the best model among the three alternatives.

### 3.3. LM Networks

Metastasis recognition requires even more attention because of its complexity. In addition, it is the mainstay of the architecture, as it provides support for examining the effect of new anticancer and antimetastatic drugs through segmentations performed at different times.

The observed performance of the LM networks was slightly lower than that of HL and MLA networks, as expected due to the higher task complexity. Indeed, the results remained good in terms of high accuracy and specificity, although some of the other metrics showed lower values. In particular, low IoU and Dice values were observed, despite the high accuracy and specificity, due to the low number of positive pixels in the GT. In fact, as documented in the literature, IoU and Dice may not be appropriate metrics for the segmentation of small structures, as a single-pixel difference between two masks can have a huge impact on these metrics [29]. Therefore, the results for this case can be better evaluated in terms of simpler metrics, such as accuracy and specificity, which were satisfactory.

Once again, none of the models outperformed in all metrics, and the same tradeoff analysis was performed. As in the MLA case, U-net-2 and U-net-3 performed best in terms of IoU and Dice coefficients.

**Figure 5:**
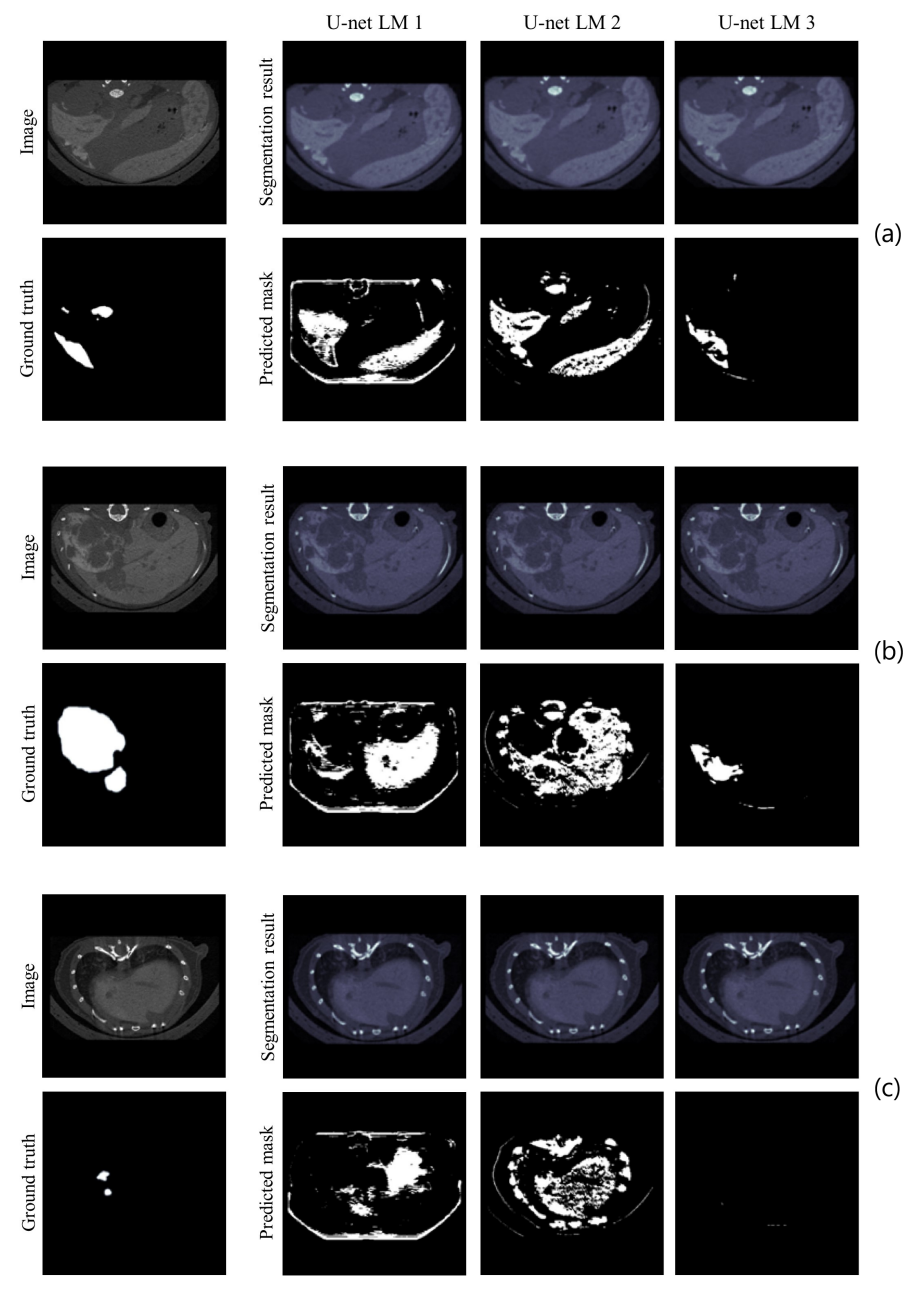
Sample images of liver with metastases for LM: proximal slice (**a**), slice in the middle (**b**), and distal slice (**c**). The acquired image, the corresponding GT, the LM segmentations before binarization, and the predicted BPMs from all networks (U-net-1, U-net-2, and U-net-3) are reported for each slice.

The same visual examination was then performed. Figure 5 shows some sample images in the validation set, the corresponding GT, the segmentation results before binarization, and the BPM. U-net-3 showed that it could recognize the main features of the metastases and exclude liver areas without metastasis in proximal and central slices, where metastases are relevant. When metastases were small, as in the distal slice, none of the models recognized them. Therefore, although BPMs do not exactly match GTs, U-net-3 appeared to appropriately exclude metastasis-free portions of the liver and wisely detect metastases and diseased liver tissue around them.

However, when looking at a central slice (Figure 5b), even U-net-3 showed some weakness in correctly recognizing metastases due to the identical gray intensity of liver tissue and metastases. This criticality is the same as that encountered by human operators, as the shades of gray are imperceptible to the naked eye, especially in areas where the edges of the liver are no longer evident. In fact, the process required sacrificing the mouse to directly observe the excised liver.

Moreover, although the accuracy was high, the predicted masks suffered from systematic errors and included the mouse spine with the marrow and the outer polystyrene structure on which the mouse lays during the micro-CT scan. To reduce this, in light of practical applicability, an alternative mask was obtained from U-net-3 (the best performing network) after manually removing the spine, the polystyrene structure, and other irrelevant areas from the images. This manual procedure involves limited effort for the operator and does not detract from the support provided by the proposed architecture.

**Figure 6:**
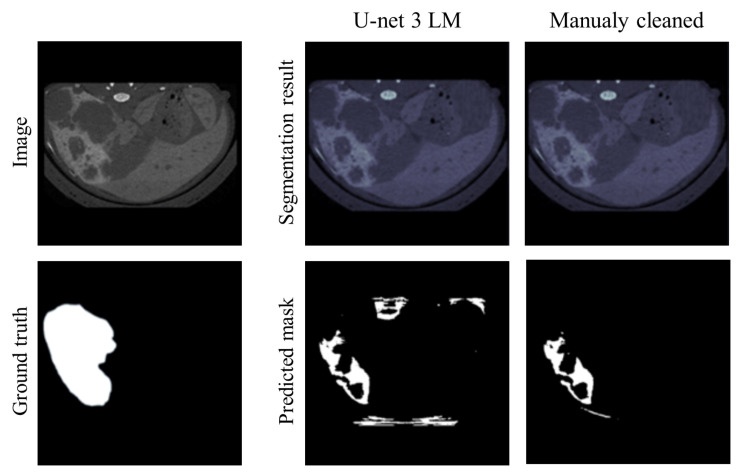
Comparison between the original U-net-3 for LM (**left**) and the alternative one based on the manually cleaned images (**right**): image and GT common to both alternatives, LM segmentation before binarization, and predicted BPM.

In this case, the original dataset was again used for the training and validation phases, while the updated dataset was used only for the prediction. Better results were achieved (Table 4), comparable to those obtained above for the simpler HL and MLA tasks. For example, slightly higher accuracy was obtained by the alternative mask with respect to the original one, equal to 91.5%. Figure 6 shows a comparison between the alternatives. On the contrary, also using the updated images for training and validation did not improve the performance.

The use of the liver mask could be a valid alternative for cleaning that could also reduce the network effort by limiting the area to be analyzed. However, in light of the final application, this would require more effort by operators who would have to apply the liver mask after checking its correctness. Therefore, we preferred the manual removal of the irrelevant areas.

### 3.4. Combined Mask

**Figure 7:**
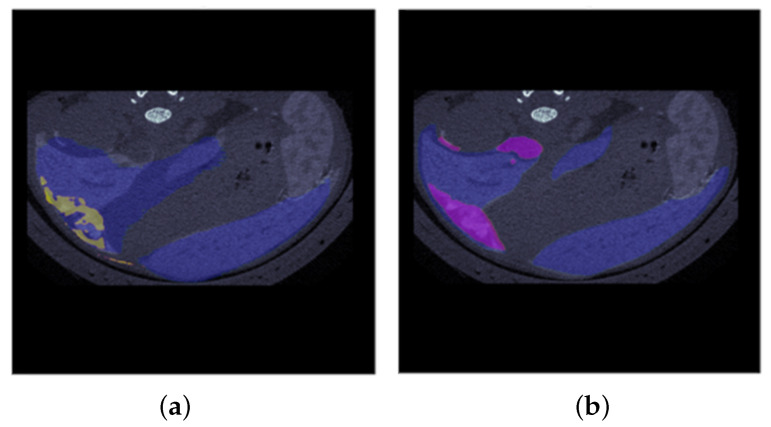
Examples of a combined mask: BPM for metastases in yellow and metastatic liver surface in blue (**a**); GT with metastases in fuchsia and metastatic liver surface in blue (**b**).

The outcomes of the MLA and LM networks were combined into merged masks that include both MLA and LM (example in Figure 7). Such visualization is useful to understand the overall appearance of the slice and to grasp the extent of metastasis within the liver.

## 4. Discussion

The obtained results allowed us to identify a network alternative that provided better results than the others for each task. None of the network alternatives outperformed the others in all metrics, and therefore a tradeoff was considered by looking at key metrics. Among them, in addition to the classical ones, NPV was also considered, because in this specific application, it is of paramount importance not to miss any positive regions in the image such as not to detect a metastatic area.

U-net-1 was identified as the best for HL and U-net-3 for MLA and LM. These best networks performed well in terms of accuracy (about 90%) and specificity (above 93%), which were comparable to those obtained by human operators and the benchmark values available in the literature, although in our pilot study these values refer to the validation set. For example, Sarvamangala and Kulkarni [30] examined the use of CNNs in medical image understanding and reported accuracies between 90% and 100%, and specificities between 80% and 98% for image segmentation tasks. Li et al. [31] also reported similar values from deep learning algorithms in medical image analysis. Bakator and Radosav [32] overviewed the applications of deep learning to medical diagnosis and reported very heterogeneous scores based on the specific application; excluding the lowest scores observed for specific and complex applications, our values are in line with those reported. Similarly, Tian et al. [33] reported accuracies of 92% and specificities of 94% for CNNs applied to CT images.

Some critical issues were observed for LM segmentation, which is the most challenging, with U-net-3, as well as the others, not correctly recognizing metastases in central slides. However, as mentioned earlier, even the experienced human operator was unable to solve this problem, and it was necessary to sacrifice the mouse to directly observe the liver and complete the manual segmentation.

Therefore, it can be considered that the proposed architecture represents a valid tool that can support the segmentation task, providing adequate results comparable to those obtained manually. However, in contrast to manual segmentation, we achieved much shorter segmentation times, which were associated with the standardization and repeatability typical of automatic segmentation. In fact, when human intervention was required, it was limited, not time-consuming, and related to easy and marginal tasks not subject to inter and intraoperator variability.

This work can be considered a pilot study, which has some limitations common to pilot studies. The first limitation concerns the amount of data. A large number of images were included in the dataset, but from a very limited number of animals. In this regard, for now, we limited the analysis to the validation set, but for full extendibility of the results, it would be appropriate to include a test set with animals not seen during training.

In addition, different disease burdens can be observed in metastatic livers: moderate if metastases are located in only part of the organ or advanced if metastases occupy a large part of the organ volume. In the pilot analysis presented here, we focused only on advanced burden cases, considering one MLA and one LM network. This choice was because, in advanced cases, there are the biggest differences from liver to liver and the problem is more complex, so it is the most critical situation to deal with. In the future, to generalize the results, additional MLA and LM networks differentiated according to metastatic burden should be considered in the proposed architecture, or the extension of a single network for each task to cope with different burdens should be considered.

Finally, the entire workflow could be fully automated, with particular reference to the first image screening, which determines whether an image belongs to a healthy or metastatic liver. Although this is a simple task even when performed manually, a classification network could be implemented to fully automatize the entire procedure, even in the presence of several metastatic burdens. This would be a simpler task than the other segmentation tasks. In fact, it would provide an overall response associated with all the image slices of an animal and not the classification of every single pixel of every single slice, as required for segmentation.

## 5. Conclusions

To train ad hoc networks to recognize PDAC liver metastases is an important requirement because of the specific geometrical characteristics of these metastases. However, only few studies have developed networks dedicated to their segmentation [17,18], while this is one of the first works dealing with metastases in the liver. More specifically, in this pilot study, we trained alternative U-net architectures to address three important segmentation tasks in preclinical mouse studies of PDAC liver metastases.

The results showed good performance, with accuracy between 88.6% and 92.6%, specificity above 93.8%, and acceptable values for the other considered metrics on the validation set for the network alternative selected for each segmentation task (HL, MLA, or LM). Moreover, we achieved reduced segmentation times compared with those of experienced human operators, and at the same time, we achieved full reproducibility because inter and intraoperator variability were avoided. Therefore, our approach can be a ready-to-use tool that speeds up work and comes to the aid of biologists during preclinical studies.

We are aware that further analyses on larger datasets are needed to fully validate the tool, but the results of this pilot study represent a promising starting point for future developments. At the same time, to move from preclinical application on mice to clinical application on patients, it is necessary to re-train the networks from scratch, possibly revising their structure.

## Figures and Tables

**Table 1:** Layers of the alternative CNNs tested.

	Network Structure		
	U-Net-1	U-Net-2	U-Net-3
Layers – contracting	Block 1	Block 2	Block 3
	2×2 max pooling	2×2 max pooling	2×2 max pooling
	Block 1	Block 2	Block 3
	2×2 max pooling	2×2 max pooling	2×2 max pooling
	Block 1	Block 2	Block 3
	2×2 max pooling	2×2 max pooling	2×2 max pooling
	Block 1	Block 2	Block 3
	2×2 max pooling	2×2 max pooling	2×2 max pooling
	Block 1	Block 2	Block 3
	2×2 max pooling	2×2 max pooling	2×2 max pooling
	Block 1	Block 2	Block 3
Layers – expanding	2×2 up convolution	2×2 up convolution	2×2 up convolution
	concatenation	concatenation	concatenation
	Block 1	Block 2	Block 3
	2×2 up convolution	2×2 up convolution	2×2 up convolution
	concatenation	concatenation	concatenation
	Block 1	Block 2	Block 3
	2×2 up convolution	2×2 up convolution	2×2 up convolution
	concatenation	concatenation	concatenation
	Block 1	Block 2	Block 3
	2×2 up convolution	2×2 up convolution	2×2 up convolution
	concatenation	concatenation	concatenation
	Block 1	Block 2	Block 3
	1×1 convolution	1×1 convolution	1×1 convolution

**Table 2:** Hyperparameters of the specialized networks.

Hyperparameter	Value		
	HL Network	MLA Network	LM Network
Number of epochs	20	20	20
Batch size	8	10	8 (U-net-1); 10 (U-net-2 and U-net-3)
Steps per epoch	35	55	65 (U-net-1 and U-net-3); 35 (U-net-2)
Number of filters	8	8	8 (U-net-1 and U-net-2); 5 (U-net-3)
Learning rate	$10^{-3}$	$10^{-1}$	$10^{-3}$
Weight initialization	Glorot	Glorot	Glorot

**Table 3:** Rates of TP, TN, FP, and FN over all pixels, expressed as percentages, and evaluation metrics obtained from all networks.

	HL			MLA			LM		
	U-Net-1	U-Net-2	U-Net-3	U-Net-1	U-Net-2	U-Net-3	U-Net-1	U-Net-2	U-Net-3
${\mathrm{TP}}_{\%}$	9.3%	11.4%	11.1%	13.5%	16.6%	16.6%	2.0%	3.6%	2.0%
${\mathrm{TN}}_{\%}$	83.3%	77.6%	79.5%	73.1%	71.8%	71.9%	79.3%	79.0%	88.3%
${\mathrm{FP}}_{\%}$	3.9%	9.6%	7.8%	3.6%	4.9%	4.8%	10.6%	10.9%	1.6%
${\mathrm{FN}}_{\%}$	3.5%	1.4%	1.7%	9.8%	6.7%	6.7%	8.1%	6.5%	8.1%
Accuracy	92.6%	89.0%	90.5%	86.7%	88.3%	88.6%	81.3%	82.6%	90.3%
Specificity	95.5%	89.0%	91.1%	95.4%	93.6%	93.8%	88.2%	87.9%	98.2%
Precision	70.4%	54.2%	58.7%	79.0%	77.2%	77.7%	16.0%	24.9%	55.6%
NPV	96.0%	98.3%	97.9%	88.2%	91.4%	91.5%	90.7%	92.4%	91.6%
Recall	72.8%	89.2%	86.6%	58.0%	71.1%	71.2%	19.9%	35.6%	19.7%
IoU	55.8%	50.9%	53.8%	50.3%	58.8%	59.2%	9.7%	17.1%	17.0%
Dice	71.6%	67.4%	70.0%	66.9%	74.0%	74.4%	17.7%	29.3%	29.1%

**Table 4:** Rates of TP, TN, FP, and FN over all pixels, expressed as percentages, and evaluation metrics obtained from the alternative LM network.

	Alternative LM
	U-Net-3
TP%	1.8%
TN%	89.8%
FP%	0.1%
FN%	8.3%
Accuracy	91.5%
Specificity	99.9%
Precision	94.7%
NPV	91.5%
Recall	17.6%
IoU	17.3%
Dice	29.9%

## Data Availability

The image datasets used in this study can be found in Zenodo online repository (https://doi.org/10.5281/zenodo.10677614).
